# A Synchronous Robotic Resection of Colorectal Cancer and Liver Metastases—Our Initial Experience

**DOI:** 10.3390/jcm12093255

**Published:** 2023-05-02

**Authors:** Yaron Rudnicki, Ron Pery, Sherief Shawki, Susanne Warner, Sean Patrick Cleary, Kevin T. Behm

**Affiliations:** 1 Department of Surgery, Division of Colon and Rectal Surgery, Mayo Clinic, Rochester, MN 55905, USA; 2 Department of Surgery, Meir Medical Center, Faculty of Medicine, Tel Aviv University, Kfar Saba 4428164, Israel; 3 Department of Surgery, Division of Hepatobiliary and Pancreatic Surgery, Mayo Clinic, Rochester, MN 55905, USA; 4 Department of Surgery and Transplantation, Sheba Medical Center, Faculty of Medicine, Tel Aviv University, Kfar Saba 4428164, Israel

**Keywords:** robotic surgery, metastatic colorectal cancer, colorectal surgery, liver surgery

## Abstract

Introduction: Synchronous robotic colorectal and liver resection for metastatic colorectal cancer (mCRC) is gaining popularity. This case series describes our initial institutional experience. Methods: A retrospective study of synchronous robotic colorectal and liver resections for metastatic colorectal cancer (March 2020 to December 2021). Results: Eight patients underwent synchronous robotic resections. The median age was 59 (45–72), and the median body mass index was 29 (20–33). Seven received neoadjuvant chemotherapy, and five rectal cancers received neoadjuvant radiotherapy. One patient had a low anterior resection with major hepatectomy, two had low anterior resection with minor hepatectomy, and one had abdominoperineal resection with major hepatectomy. One patient had a left colectomy with minor hepatectomy, and two had right colectomies with minor hepatectomy. We used five robotic 8/12 mm ports in all cases. Extraction incisions were Pfannenstiel in four patients, colostomy site in two patients, one perineal incision, and one supra-umbilical incision. The median estimated blood loss was 200 mL (25–500), and the median operative time was 448 min (374–576). There were no intra-operative complications or conversions. Five patients had the liver resection first, and two of six anastomoses were performed before the liver resection. The Median length of stay was 4 days (3–14). There were two post-operative complications, prolonged ileus and DVT, with a Clavien-Dindo complication grade of I and II, respectively. There were no readmissions or reoperations. All colorectal and liver resection margins were negative. Conclusions: Synchronous robotic colorectal and liver resection can be performed effectively utilizing one port configuration with acceptable short-term outcomes and quality of oncologic resection.

## 1. Introduction 

Colorectal cancer (CRC) is the third most common cancer worldwide, and the fourth most common cause of cancer mortality is primarily attributed to CRC metastases (mCRC) [1]. A quarter of newly diagnosed CRC patients are diagnosed yearly with a stage IV metastatic disease, mainly metastases to the liver [2,3]. Treatment for stage IV CRC disease is usually a combination of treatments with systemic chemotherapy, possible immunotherapy, possible chemoradiation for rectal cancer, and surgery when appropriate [4,5]. The rate of patients with CRC and liver metastasis amenable to surgery has risen substantially over the last decade, with multiple large epidemiological studies demonstrating an incidence of 14–17% of patients who present with synchronous liver metastases, which occur more frequently in male patients [6]. Most often, the surgical plan is based on a staged approach, either colorectal resection first followed by liver resection at a second stage or the other way around, most often with systemic or local treatment in between. When assessing the liver disease burden, the traditional treatment is resection when there is a resectable disease with or without perioperative systemic treatment, usually a combination of chemotherapy and molecular treatment such as Avastin. When there is a non-resectable liver disease, other forms of localized treatments can be considered, such as ablation or irradiation. In a select group of patients, a synchronous colorectal resection combined with partial liver resection is a viable and preferable surgical option. For some patients, the burden of a combined open surgery can be mitigated by a synchronous minimally invasive approach [7].

The advantages of a synchronous minimally invasive CRC and liver resection are reduced blood loss, early mobility, lower rates of surgical site infections, shorter length of stay, and reduced time interruption of systemic therapy. These peri-operative and oncologic benefits can be combined with the advantages of robotic surgery, mainly three-dimensional augmented vision, the flexibility of wristed instruments, tremor suppression, and better access to the posterosuperior segment’s lesions [8,9]. Robotic colorectal resection has become prevalent in the Western world, yet not many centers perform robotic liver resection, let alone synchronous robotic colorectal and liver surgery. Although synchronous robotic colorectal and liver resection for mCRC is gaining popularity, very little was published on this approach’s feasibility, safety, and manner. This case series describes our initial institutional experience with a synchronous robotic approach and offers some recommendations regarding how we do it.

## 2. Materials and Methods 

A retrospective study was designed to identify patients that underwent a combined synchronous robotic colorectal and liver resection for metastatic colorectal cancer at the Mayo Clinic. This study was conducted in accordance with the ethical principles of the Declaration of Helsinki (Edinburgh 2000) and the approval of the Institutional Review Board. A prospectively maintained institutional database was queried for patients that underwent this procedure from March 2020 to December 2021. Data collected included demographic information such as age, gender, Body mass index (BMI), type of liver and colorectal resection, number of robotic ports used, specimen incision extraction site, length of operation, intraoperative complications, and estimated blood loss, and preoperative neoadjuvant treatment.

**Figure 1:**
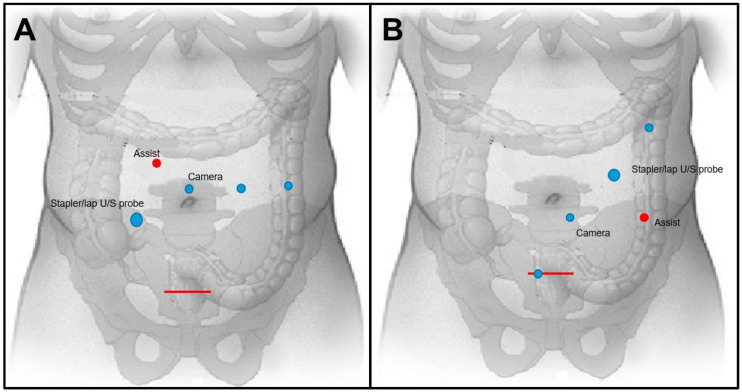
Diagram of two robotic port placement arrays for combined colorectal and liver resection. (**A**) Horizontal line array at the level of the umbilicus for rectal and sigmoid colon resection, red line marks a Pfannenstiel incision. (**B**) Diagonal line array from the left upper abdominal quadrant to the right lower quadrant for right colon resection.

All cases were performed using the da Vinci XI platform. After laparoscopic exploration of the abdomen, all patients had four robotic trocars inserted, three 8 mm trocars and one 12 mm trocar, and another 8 mm AirSeal assistant trocar. The robotic port arrays and positions over the abdominal wall were mainly in a horizontal line at the level of the umbilicus for the rectal and sigmoid cases and with a slight diagonal line from the left upper quadrant to the right lower quadrant for the cases with a right colon lesion (Figure 1). Therefore, there was no need for any added ports or repositing of ports for the liver resection.

In addition, the patient’s post-operative course was monitored for the length of stay, post-operative complications, and pathological reports, including resection margins. The procedures were performed by three dedicated colorectal surgeons and three dedicated hepatobiliary surgeons. The order of resection and timing of anastomosis creation was decided by each colorectal and hepatobiliary surgeon’s preference. The definition of a major hepatectomy was defined as a complete resection of three or more continuous liver segments and a minor hepatectomy was defined as any resection that included less than the above.

## 3. Results

During a period of 22 months, eight patients with metastatic colon or rectal cancer (CRC) with liver metastasis underwent synchronous robotic resections. The patient cohort comprised four males and four females, with a median age of 59 years with a range of 45–72 and a median body mass index (BMI) of 29 kg/m^2^ with a range of 20–33 kg/m^2^. Five patients were diagnosed with rectal cancer, four in the mid-rectum and one in the low rectum. One patient was diagnosed with sigmoid colon cancer and two with ascending colon cancer. All patients had CRC metastases to the liver that were initially diagnosed as resectable. Five had solitary metastases, one had two bilobar metastases, one had three unilobar metastases, and one had three bilobar metastases. All liver lesions other than one were relatively peripheral, which allowed for minor hepatectomies. No metastases involved the major portal or venous pedicles. Two patients were obstructed and initially underwent loop colostomy creation before neoadjuvant treatment. Perioperatively, seven patients (87.5%) received neoadjuvant chemotherapy using a FOLFOX protocol, with three receiving Bevacizumab. All five rectal cancer patients received neoadjuvant radiotherapy, four had a short course, and one had a long course of radiotherapy. One patient with an ascending colon lesion and a solitary 4b/5 liver metastasis went straight to surgery with no neoadjuvant treatment (Table 1).

### 3.1. Operative Management

Out of the five patients with rectal cancer, four underwent robotic low anterior resection (rLAR), one with a loop colostomy closure. Out of these, one underwent a synchronous robotic left hepatectomy, and the other four had minor hepatectomies with segmental, subsegmental, or wedge liver resections. All four rLAR patients also had a diverting loop ileostomy (DLI) created, and two female patients also had a bilateral salpingo-oophorectomy (BSO), one of them with a total abdominal hysterectomy (TAH) and a partial upper vaginectomy. One patient underwent a robotic abdominoperineal resection (APR) and an end colostomy creation, with a minor hepatectomy and segment 2 and 3 sub-segmentectomy. One patient had a left colectomy for a proximal sigmoid lesion, loop colostomy closure with a primary anastomosis, and a minor hepatectomy, segment 3 resection. Two patients with ascending colon cancer underwent right colectomies with primary anastomoses and minor hepatectomies (Table 2).

The six rectal and sigmoid cases required a two quadrants approach, repositioning the robotic boom once when transitioning between the rectal and liver resection. The two right colectomies did not require repositing the robotic boom and utilized a single abdominal quadrant approach. In the hepatectomy part, there were no hilar dissections or need for the Pringle maneuver. Parenchymal transection was performed with a combination of unipolar diathermy, bipolar diathermy, and a vessel sealer.

The liver resection was performed first, followed by the colorectal resection in five patients (63%). In two rectal resections and one colonic resection, the colorectal resection was performed first, followed by the liver resection, with the two colorectal anastomoses performed before the hepatic portion and the ileocolic anastomosis created after the hepatic portion was completed. Following the completion of both resections and reconstructions, the specimens were extracted through a Pfannenstiel incision in four patients, a colostomy site in two patients, a perineal incision in the APR case, and one through a limited supra-umbilical incision. There were no intraoperative complications, and none needed any conversion to an open approach. The median estimated blood loss was 200 mL (25–500), and the median operative time was 448 min (374–576) (Table 3).

### 3.2. Post-Operative Period and Outcomes

The median length of stay of patients was 4 days, with a range of 3 to 14 days. Two of the eight patients (25%) had post-operative complications, with one patient having a post-operative ileus and one developing deep vein thrombosis (DVT), with a Clavien-Dindo post-operative complication grade of I and II. There were no cases of readmissions or reoperations.

Final pathology reports showed negative margins on all colorectal and liver resections, with six colorectal lesions having a penetration level of T3 and two of T4. Four patients were found to have positive lymph nodes in the mesentery, and four patients had no positive nodes found, the number of lymph nodes harvested ranged from 16 to 49. The average size of the hepatic lesions was 1.9 ± 1.3 cm, with lesions ranging from 0.5 cm to 4.5 cm. No post-operative mortality was reported through an average follow-up time of 29 ± 20 months (Table 4).

## 4. Discussion

In this case series, we report our experience performing combined synchronous robotic colorectal and liver resections for mCRC. The surgical treatment of stage IV mCRC patients is still evolving, with no clear answer to the optimal strategy. The burden of systemic treatment with two major surgeries is challenging for some patients and can sometimes be mitigated by performing a combined minimally invasive surgical approach [6,10]. Presumably, the robotic approach allows for more flexibility in combining both CRC surgery and liver metastasis surgery compared to the laparoscopic approach, with improved visualization in a three-dimensional view, and wristed instruments that allow better reach and control in the pelvis for rectal lesions and over the liver for “hard to reach” liver lesions, so that a preferred outcome can be attained [11,12]. These assumptions are the results of small single-institution series, and the literature to support these assumptions is still lacking.

From a colorectal point of view, there is some difference between a right colon resection, a left or a sigmoid colon resection, and rectal resection. The difference is mainly in the added complexity of the rectal resection that requires a pelvic dissection, sometimes after neoadjuvant pelvic radiation that brings upon a protective diverting loop ileostomy. As an institutional practice, all patients with known metastatic rectal disease are generally referred to a short course of radiation in an effort to shorten treatment duration and minimize time off of systemic therapy prior to surgery. The duration of radiation is dictated by the morphology of the primary rectal cancer both at presentation and after neoadjuvant chemotherapy. For patients with threatened CRM, T4 tumors, and tumors with clinically positive extra-mesorectal lymph nodes, a long course of radiation is preferred. Another difference is the robotic ports array, which is more often a personal choice of the CRC and Liver surgeons. In this series, we used five trocars and positioned them over the abdominal wall in a horizontal line at the level of the umbilicus for the rectal and sigmoid cases and with a slight diagonal line from the left upper quadrant to the right lower quadrant for the cases with a right colon lesion as seen in Figure 1.

While robotic liver resection is gaining popularity, the vast majority of liver resections worldwide are still being done using an open approach. Given safety issues and the technical complexity of proper oncologic resections deep in the liver, or in the higher and posterior segments, most laparoscopic liver resections are being performed for peripheral lesions in favorable locations. With improved articulation, a robotic approach may offer better access to otherwise challenging locations such as segments 1, 7, and 8 [13]. Therefore, many patients with limited liver metastases could be potential candidates for robotic resections, as seen in the meta-analysis of Rocca et al. with a 131 robotic mCRC liver resection [14]. There are very few published series of synchronous robotic colorectal resection combined with liver resection for metastatic colorectal cancer. A recent meta-analysis by McGuirk et al. collected only 28 patients that underwent this simultaneous robotic approach. They showed a similar average operative time and a similar low rate of complications, but a hospital length of stay that was more than double in our cohort [15].

From a technical point of view, in a synchronous right colon and liver resection, the robotic platform is usually used in a single right upper quadrant orientation, which does not necessitate changes in the robotic arms array [16]. On the other hand, one of the challenges in a synchronous rectal and liver resection is the multi-quadrant approach and the need to “boom around” from a robotic pelvic dissection of the rectum to a right upper quadrant dissection for the liver and repositioning of the robotic tools. However, in a well-trained and dedicated robotic surgery team, this change in the robot position does not require more than a few minutes.

The optimal sequence of resection, colorectal first followed by liver resection or the other way around, is debatable, with advantages and disadvantages for each approach. The colorectal resection requires manipulation of the small bowel for exposure. It may be considered the “contaminated” part of the surgery with bowel resection. In contrast, the liver resection may necessitate portal vein occlusion (the Pringle maneuver) which can lead to bowel edema and low central venous pressure which can lead to hypotension and hypoperfusion. In our series, both sequences were employed based on the patient’s disease specification, mainly the complexity of the liver resection part and the surgeon’s preference. In addition, some thoughts were given to the timing of the bowel reconstruction by reserving the anastomosis creation to the final part of the surgery to avoid challenging the blood flow to the anastomosis while performing the liver resection.

In minimally invasive surgery and robotic surgery specifically, the specimens’ extraction site can be chosen in a manner that will avoid a midline incision and a high risk for post-operative hernia and other wound complications. In this study, most specimens were taken out through a Pfannenstiel incision, a colostomy site incision, or through the perineal incision, with only one case needing a limited midline incision. With dedicated CRC and liver robotic surgeons and teams, the synchronous robotic approach can be performed with safety and relative ease, with limited intraoperative complications, no need for conversion to an open approach, and limited blood loss, as seen in this study. In the future, we believe that the robotic platform may be the platform of choice for more challenging cases like carcinomatosis management.

This study’s limitations revolve around its small sample size, the heterogeneous mix of mCRC cases, and its retrospective nature. It might be challenging to extrapolate from this series for cases in other centers, yet, due to the limited number of cases published in the literature, no randomized control studies in the near future, and very few centers in the world that can perform a synchronous robotic colorectal and liver resection, this series can lay the groundwork for establishing these capabilities for future cases and show what is safe and feasible. Further studies should be planned and executed to prove its advantage to patients with mCRC. Further studies with larger cohorts and longer follow-ups are needed to assess the long-term outcomes of robotic combined resections.

## 5. Conclusions

Synchronous robotic colorectal and liver resection of colorectal cancer and liver metastases is a safe and feasible approach for select patients with liver mCRC. This approach can be performed effectively utilizing one port configuration with acceptable short-term outcomes and quality of oncologic resection. However, further studies are needed to assess the benefits of the robotic approach, especially compared to laparoscopy and open surgery.

## Figures and Tables

**Table 1:** Baseline Demographic, location of lesions, and neoadjuvant treatment.

Characteristic	n = 8
Age (years)—median (range)	59 (45–72)
Female sex—n (%)	4 (50%)
Body mass index (BMI) kg/m^2^—median (range)	29 (20–33)
Location of CRC lesion	
Rectal cancer—n	5
Sigmoid colon cancer—n	1
Ascending colon cancer—n	2
Location of metastatic spread in the liver	
One metastasis—Right lobe	2
One metastasis—Left lobe	3
Two metastases—Bilobar	1
Three metastases—Left lobe	1
Three metastases—Bilobar	1
Prior loop colostomy creation for colonic obstruction—n	2
Neoadjuvant chemotherapy—n (%)	7 (87.5%)
Added neoadjuvant Bevacizumab—n (%)	3 (37.5%)
Neoadjuvant chemoradiotherapy for rectal cancer—n (%)	5 (100%)

**Table 2:** Operative management.

Characteristic	n = 8
Type of colorectal procedure	
Robotic low anterior resection (rLAR) with DLI—n	4
Robotic abdominoperineal resection (APR)—n	1
Robotic left colectomy—n	1
Robotic right colectomy—n	2
Type of liver resection	
Major hepatectomy	2
Minor Hepatectomy	6
Robotic ports array over the abdominal wall	
Horizontal line at the level of the umbilicus—n	6
Diagonal line from LUQ to RLQ—n	2
Liver resection performed first—n	5
Colorectal resection performed first—n	3
Colorectal anastomoses performed before the hepatic resection	2
Specimens’ extraction site	
Pfannenstiel incision—n	4
Colostomy incision—n	2
Perineal incision after APR—n	1
Limited supra-umbilical midline incision—n	1
Intraoperative complications—n	0
Conversion to an open approach—n	0
Estimated blood loss—ml-median (range)	200 (25–500)
Operative time—minutes-median (range)	448 (374–576)
DLI—Diverting loop ileostomy	
LUQ—Left upper quadrant	
RLQ—Right lower quadrant	
ml—Milliliter	

**Table 3:** Patients list with the type of robotic colorectal and liver surgery and operative time.

Raw	Age/Gender	Colorectal Surgery	Liver Surgery	Operative Time (Hours)
1	56 M	Robotic abdominoperineal resection (APR)	Robotic left hepatectomy	6:18
2	45 M	Robotic low anterior resection (LAR)	Robotic partial hepatectomy (segments 3 + 4b)	9:36
3	72 F	Robotic right colectomy	Robotic partial hepatectomy (segments 4b + 5) & cholecystectomy	6:42
4	54 M	Robotic low anterior resection (LAR)	Robotic left hepatectomy	6:14
5	62 F	Robotic low anterior resection (LAR)	Robotic partial hepatectomy (segment 8) & cholecystectomy	8:24
6	63 M	Robotic sigmoid colectomy	Robotic partial hepatectomy (segment 3)	7:46
7	55 F	Robotic low anterior resection (LAR)	Robotic partial hepatectomy (segments 2 + 3 + 8) + ablation of liver lesion	8:34
8	62 F	Robotic right colectomy	Robotic partial hepatectomy (segments 2) + ablation of liver lesion	7:10

**Table 4:** Post-operative outcome and pathological characteristics.

Characteristic	n = 8
Length of post-operative hospital stay (LOS)—days-median (range)	4 (3–14)
Post-operative complications—n (%)	2 (25%)
Ileus	1
Deep vein thrombosis (DVT)	1
Clavien-Dindo postoperative complication grade I/II	2
Readmissions or reoperations—n	0
Positive resection margins—n	0
Level of colorectal tumor penetration	
T3—n	6
T4—n	2
Positive mesenteric lymph nodes—n	4
Size of the hepatic lesions—cm—mean ± SD (range)	1.9 ± 1.3 (0.5–4.5)
Post-operative mortality—n	0
Follow-up time—months—mean ± SD	29 ± 20
S.D.—Standard deviation	
cm—Centimeter	

## Data Availability

Data will be available upon request from authors.
